# Supplementary material for: Extensive screening reveals previously undiscovered aminoglycoside resistance genes in human pathogens
Source: Commun Biol. 2023 Aug 3;6:812. doi: 10.1038/s42003-023-05174-6 (PMC10400643; doi:10.1038/s42003-023-05174-6)
Supplement: Supplementary file 2 — Supplementary Information [file 42003_2023_5174_MOESM2_ESM.pdf]

Supplementary Information

Extensive screening reveals previously undiscovered aminoglycoside resistance genes in human pathogens

David Lund[1,2], Roelof Dirk Coertze[2,3], Marcos Parras-Moltó[1,2], Fanny Berglund[2,3], Carl-Fredrik Flach[2,3], Anna Johnning[1,2,4], D.G. Joakim Larsson[2,3], Erik Kristiansson[1,2,*]

[1]Department of Mathematical Sciences, Chalmers University of Technology and University of Gothenburg, Gothenburg, Sweden

[2]Centre for Antibiotic Resistance Research (CARe), University of Gothenburg, Gothenburg, Sweden

[3]Department of Infectious Diseases, Institute of Biomedicine, Sahlgrenska Academy, University of Gothenburg, Gothenburg, Sweden

[4]Department of Systems and Data Analysis, Fraunhofer-Chalmers Centre, Gothenburg, Sweden

Corresponding author:
Erik Kristiansson: erik.kristiansson@chalmers.se

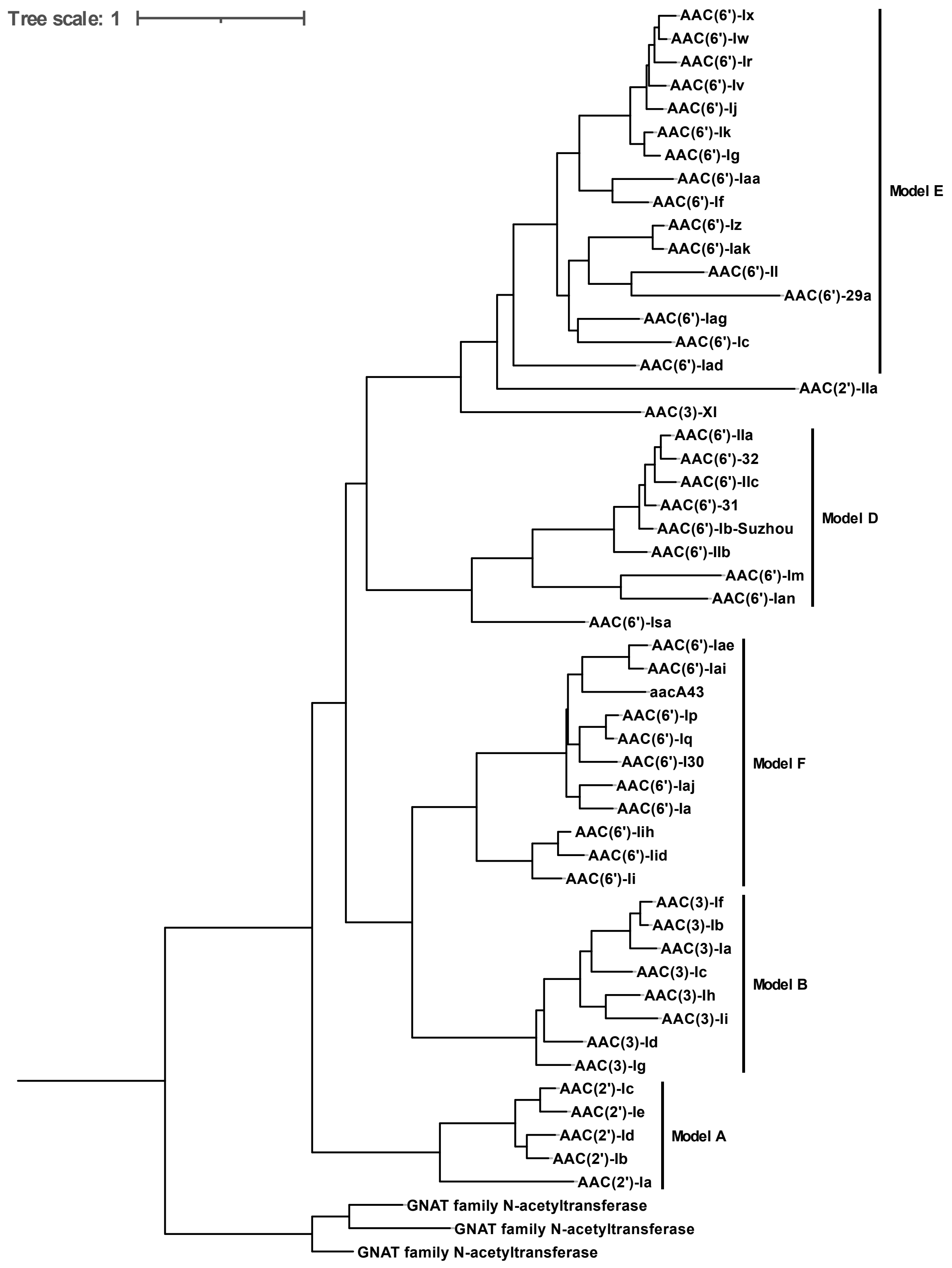

Supplementary Figure 1. Phylogenetic tree displaying known AAC sequences from the GNAT-family of N-acetyltransferases. The tree was built from centroid sequences after clustering at 90% amino acid identity. The five groups of sequences that were used to create separate models A–B, D–F are marked in the tree.

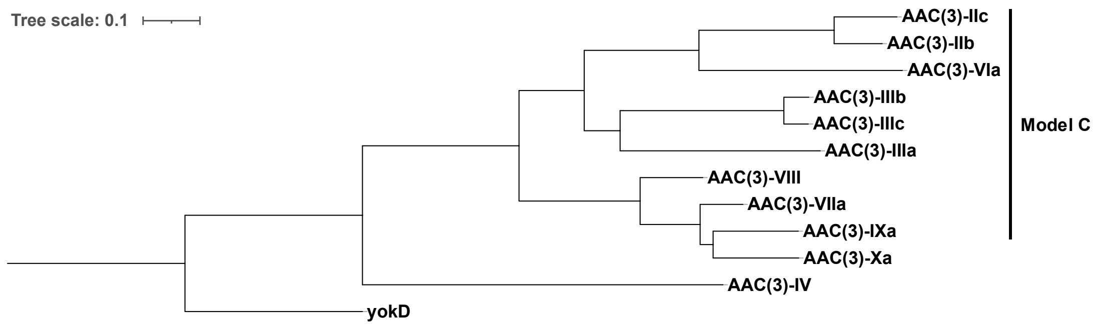

Supplementary Figure 2. Phylogenetic tree displaying known, non-GNAT like AAC(3) sequences. The tree was built from centroid sequences after clustering at 90% amino acid identity. The sequences that were used to create model C are marked in the tree.

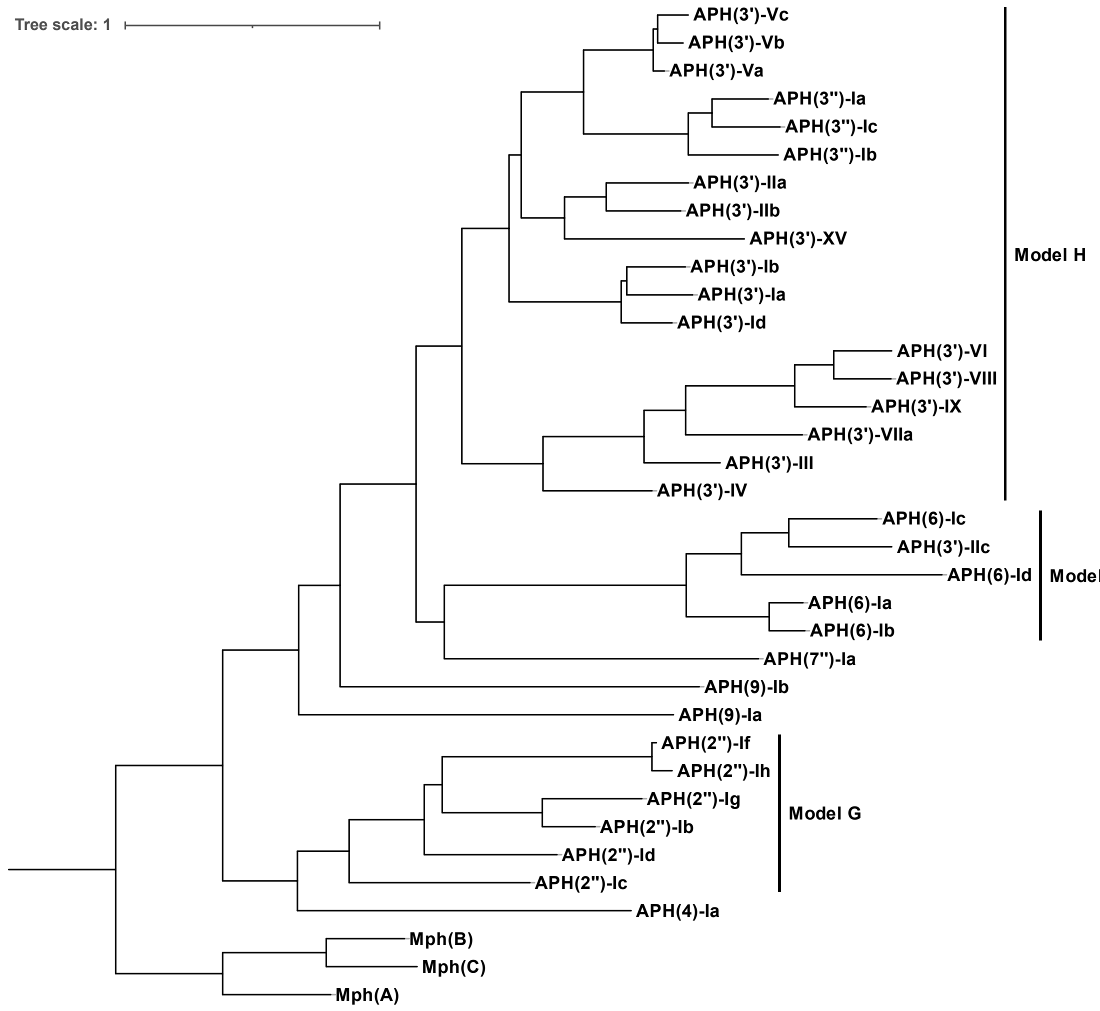

Supplementary Figure 3. Phylogenetic tree displaying known APH sequences. The tree was built from centroid sequences after clustering at 90% amino acid identity. The three groups of sequences that were used to create separate models G–I are marked in the tree.

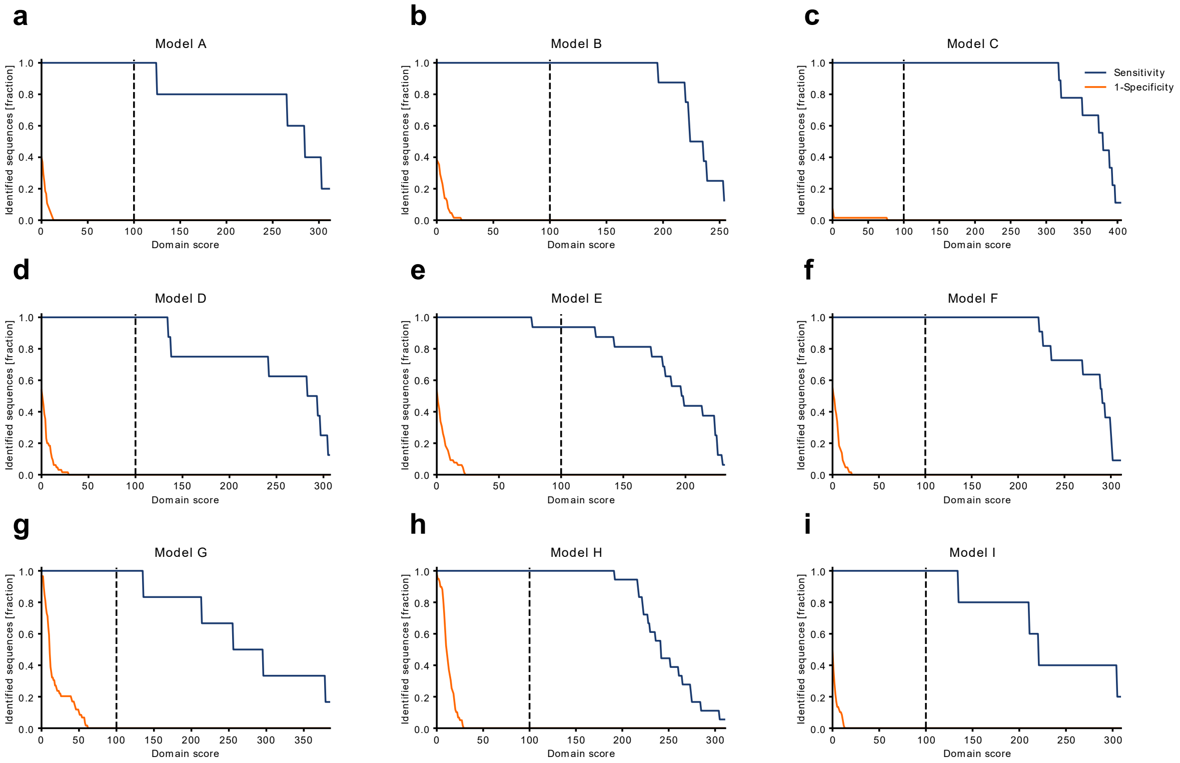

Supplementary Figure 4. Performance of the created HMMs for classifying full-length genes. Panels **a**-**i** show the sensitivity and specificity of models A-I as a function of domain score. Dashed black lines represent the optimized threshold scores used for analysis with the models.

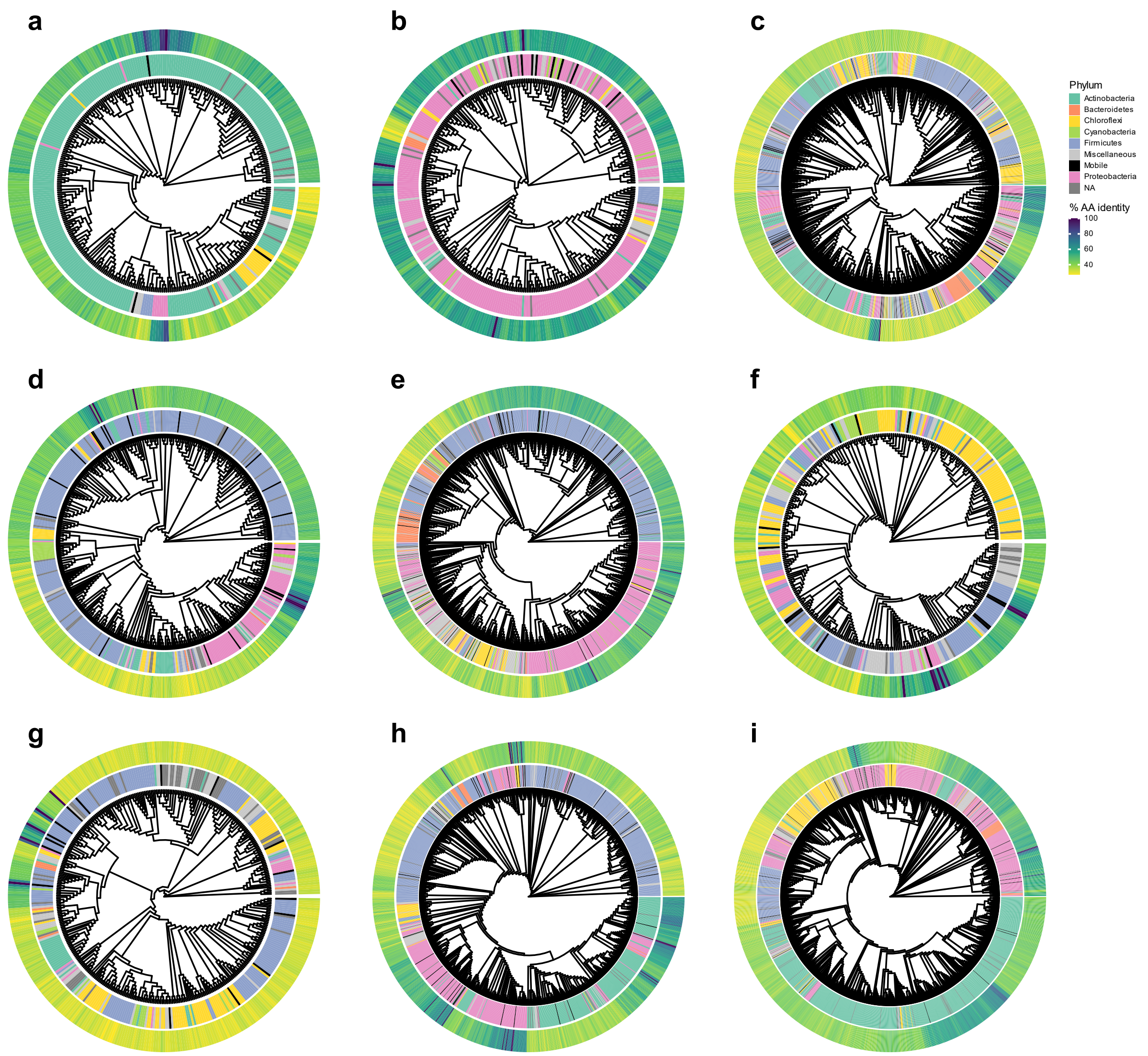

Supplementary Figure 5. Phylogenetic trees depicting the aminoglycoside resistance enzymes predicted in this study. Each tree was built from the centroid sequences after clustering at 70% amino acid identity. The colours of the inner circle indicate the phylum of the host bacteria that make up the cluster represented by each leaf. The colour black indicates that bacteria from multiple phyla are present within the same cluster. The colours in the outer circle indicate the similarity (% amino acid identity) between the representative centroid sequence of each cluster and the most similar known aminoglycoside resistance gene. Each panel depicts the AMEs predicted by a distinct model: **a** Model A (AAC(2')). **b** Model B (AAC(3)). **c** Model C (AAC(3)). **d** Model D (AAC(6')). **e** Model E (AAC(6')). **f** Model F (AAC(6')). **g** Model G (APH(2'')). **h** Model H (APH(3')+APH(3'')). **i** Model I (APH(6)+APH(3')).

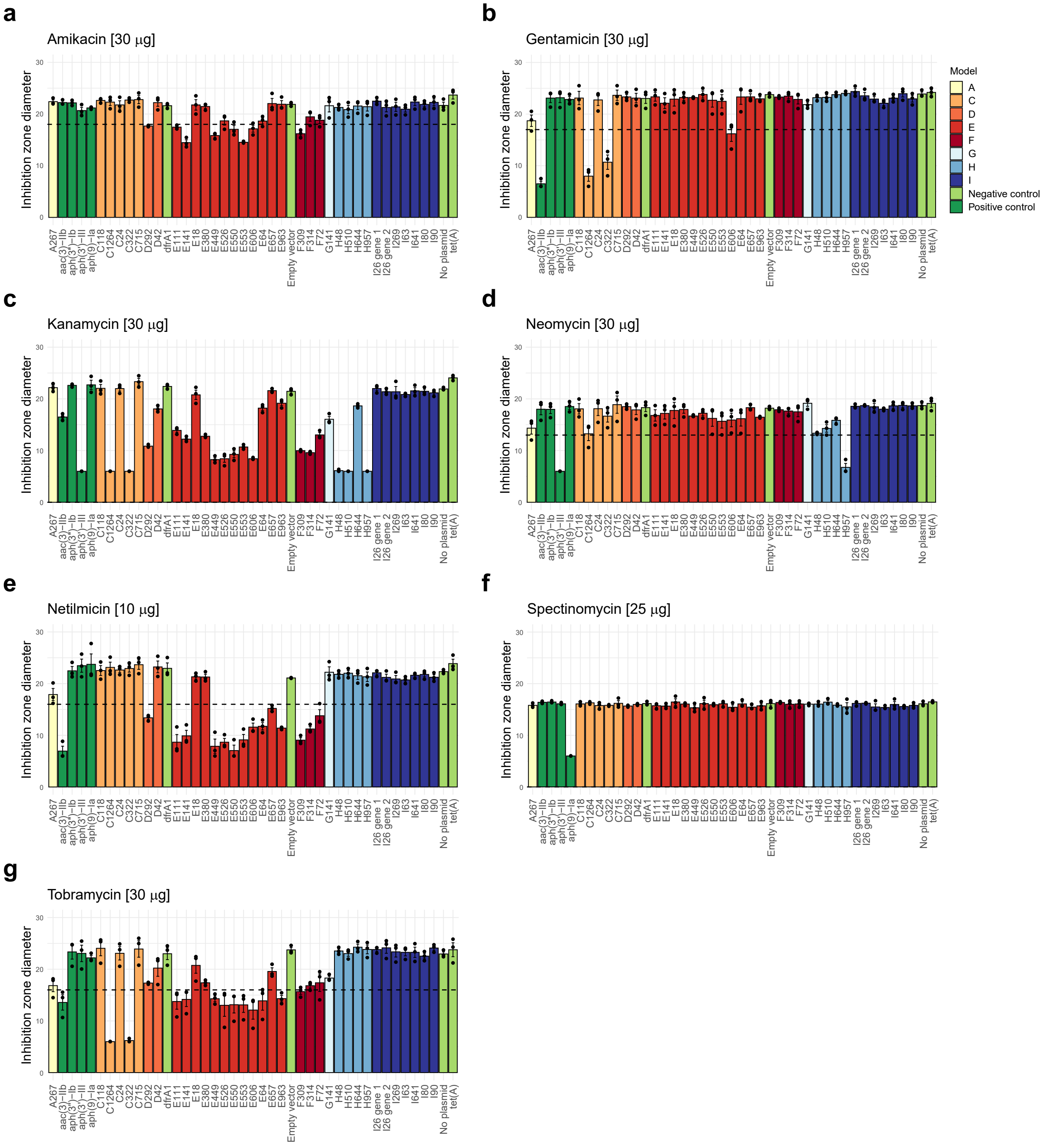

Supplementary Figure 6. **Mean zone diameters [mm] observed during disk diffusion tests of predicted new aminoglycoside resistance genes in *E. coli*.** The mean diameters were calculated from three replicates per tested gene and antibiotic (individual data points are shown in the plot). Standard deviations are displayed as error bars. Clinical breakpoints and/or epidemiological cut-off values (ECOFFs) taken from the EUCAST database (when available) are represented as dashed black lines. **a** Amikacin [30 µg]. **b** Gentamicin [30 µg]. **c** Kanamycin [30 µg]. **d** Neomycin [30 µg]. **e** Netilmicin [10 µg]. **f** Spectinomycin [25 µg]. **g** Tobramycin [30 µg].

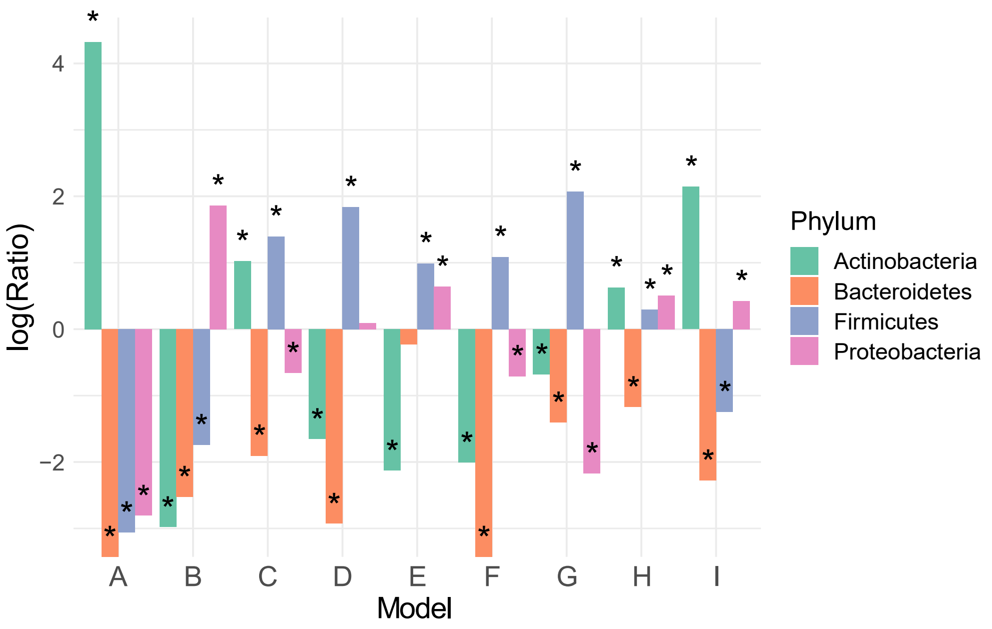

Supplementary Figure 7. Enrichment analysis of bacterial phyla harbouring an over- or under-representation of aminoglycoside resistance genes. The ratios and their significance were calculated using Fisher's exact test, a star is used to denote significant results (p<0.01).

Supplementary Table 1. Sensitivity and specificity of the created profile HMMs at the optimized threshold score.

Model	Reference phenotype(s)	Reference sequences	Sensitivity	Specificity
A	AAC(2')-I	5	1.000	1.000
B	AAC(3)-I	8	1.000	1.000
C	AAC(3)-II, III, VI, VII, VIII, IX	9	1.000	1.000
D	AAC(6')-I, II	8	1.000	1.000
E	AAC(6')-I	16	0.9375	1.000
F	AAC(6')-I	11	1.000	1.000
G	APH(2'')-I	6	1.000	1.000
H	APH(3')-I, II, III, IV, V, VI, VII, VIII, IX, XV + APH(3'')-I	18	1.000	1.000
I	APH(6)-I + APH(3')-II	5	1.000	1.000
