# Supplementary material for: Extensive screening reveals previously undiscovered aminoglycoside resistance genes in human pathogens
Source: Commun Biol. 2023 Aug 3;6:812. doi: 10.1038/s42003-023-05174-6 (PMC10400643; doi:10.1038/s42003-023-05174-6)
Supplement: Supplementary file 3 — Description of Supplementary Materials [file 42003_2023_5174_MOESM3_ESM.docx]

**Description of Additional Supplementary Files**

**File name:** Supplementary Data 1

**Description**: List of the aminoglycoside resistance genes that were used to create the Hidden Markov models.

**File name**: Supplementary Data 2

**Description:** List of new aminoglycoside resistance genes predicted in this study.

**File name**: Supplementary Data 3

**Description**: Descriptions of predicted new mobile gene families carried by pathogenic species.

**File name:** Supplementary Data 4

**Description:** p-values produced by comparing Page 8 of 23 the inhibition zone diameter of each tested new gene and antibiotic to negative controls using t-tests.

**File name:** Supplementary Data 5

**Description**: Raw data from the disk diffusion tests.

**File name:** Supplementary Data 6

**Description**: The source data behind the graphs in the paper.
